# IFNβ Is a Potent Adjuvant for Cancer Vaccination Strategies

**DOI:** 10.3389/fimmu.2021.735133

**Published:** 2021-09-06

**Authors:** Katherine M. Audsley, Teagan Wagner, Clara Ta, Hannah V. Newnes, Anthony C. Buzzai, Samantha A. Barnes, Ben Wylie, Jesse Armitage, Tsuneyasu Kaisho, Anthony Bosco, Alison McDonnell, Mark Cruickshank, Vanessa S. Fear, Bree Foley, Jason Waithman

**Affiliations:** ^1^Telethon Kids Institute, The University of Western Australia, Nedlands, WA, Australia; ^2^Department of Microbiology and Immunology, The University of Melbourne at the Peter Doherty Institute for Infection and Immunity, Melbourne, VIC, Australia; ^3^Department of Experimental Dermatology, University of Magdeburg, Magdeburg, Germany; ^4^Department of Immunology, Institute of Advanced Medicine, Wakayama Medical University, Wakayama, Japan; ^5^School of Biomedical Sciences, The University of Western Australia, Nedlands, WA, Australia

**Keywords:** type I interferon, IFNβ, cancer vaccination, adjuvant, cross-priming, CD8+ T cells, checkpoint blockade, immunotherapy

## Abstract

Cancer vaccination drives the generation of anti-tumor T cell immunity and can be enhanced by the inclusion of effective immune adjuvants such as type I interferons (IFNs). Whilst type I IFNs have been shown to promote cross-priming of T cells, the role of individual subtypes remains unclear. Here we systematically compared the capacity of distinct type I IFN subtypes to enhance T cell responses to a whole-cell vaccination strategy in a pre-clinical murine model. We show that vaccination in combination with IFNβ induces significantly greater expansion of tumor-specific CD8^+^ T cells than the other type I IFN subtypes tested. Optimal expansion was dependent on the presence of XCR1^+^ dendritic cells, CD4^+^ T cells, and CD40/CD40L signaling. Therapeutically, vaccination with IFNβ delayed tumor progression when compared to vaccination without IFN. When vaccinated in combination with anti-PD-L1 checkpoint blockade therapy (CPB), the inclusion of IFNβ associated with more mice experiencing complete regression and a trend in increased overall survival. This work demonstrates the potent adjuvant activity of IFNβ, highlighting its potential to enhance cancer vaccination strategies alone and in combination with CPB.

## Introduction

Immunotherapy has emerged in recent years as a new pillar of cancer treatment, revolutionizing outcomes for cancer patients. A variety of strategies have been developed, many of which harness T cell immunity to recognize and eliminate cancer. One such strategy utilizes therapeutic cancer vaccines capable of generating robust anti-tumor T cell responses that improve cancer control (1). Traditional vaccination protocols target tumor-associated antigens involved in tissue differentiation or antigens commonly overexpressed in cancer cells, demonstrating modest clinical success (2). Vaccines targeting recurring somatic mutations, with *KRAS*-vaccines as an example (3), have also been reported. More recently, personalized, tumor-specific vaccines (2, 4) have been developed that target immunogenic neoantigens predicted from each patients’ unique somatic mutation profile (2, 4). Two independent phase I clinical trials have demonstrated that peptide vaccinations targeting neoantigens in combination with CPB are a feasible, safe, and effective treatment strategy against melanoma (1, 4). The advances in neoantigen discovery, and the potential for synergy with CPB, have revitalized interest in the development of effective vaccination strategies for the treatment of cancer. However, to realize the full potential of cancer vaccination, optimization of the components of vaccine protocols is required, including vaccine formulation, delivery vehicles and immune adjuvants.

One promising candidate adjuvant are the type I interferons (IFNs), a family of pleiotropic cytokines first discovered for their role in inducing strong anti-viral immunity (5). Type I IFNs have also been demonstrated to possess potent anti-cancer properties (6), attributed to their direct anti-proliferative effect on tumor cells (7, 8), as well as their immunomodulatory effects (9). Indeed, type I IFNs have been shown to mediate both endogenous and treatment-induced tumor control *via* immune-dependent mechanisms (10). This function of type I IFNs can be attributed to the demonstrated effects they exert on a multitude of immune cell populations, including natural killer (NK) cells (11, 12), T cells (13, 14), B cells (15) and dendritic cells (DCs) (16–21). Of particular relevance to cancer vaccination, type I IFNs act on DCs to promote cross-priming of CD8^+^ T cells (19, 20), highlighting their potential as potent vaccine adjuvants.

While previous studies have established the capacity for type I IFNs to enhance cross-priming (16, 19, 20, 22, 23), the role of individual type I IFN subtypes remains unknown. The type I IFN family comprises 13 or 14 IFNα genes (in human and mouse, respectively) and a single IFNβ gene, as well as the lesser known IFNε, IFNκ, and IFNω subtypes (24). To date, only human IFNα2 has been used clinically for the treatment of cancer (25), with direct comparisons between subtypes rare (26). Despite signaling through the common type I IFNα/β receptor (IFNAR) (27), murine type I IFN subtypes appear to show divergent biological activities in a viral context (28) and in pre-clinical melanoma models (29). Here, we systematically screened the adjuvant potential of seven type I IFN subtypes in a whole-cell cancer vaccine model. Between these subtypes, we observed significant differences in their ability to modulate T cell function and identified IFNβ as a superior novel adjuvant that might be combined with anti-PD-L1 CPB. Our findings establish that type I IFN subtypes display divergent therapeutic activities and highlight IFNβ as an attractive candidate adjuvant for use with cancer vaccination and CPB.

## Materials and Methods

### Cell Lines

B16-F1 (B16) murine melanoma cells were purchased from the ATCC. The B16.Kb^loss^ cell line was a kind gift from Esteban Celis, University of Southern Florida, USA. B16 cells were passaged routinely at 70-80% confluency and cultured in RPMI media (Life Technologies) supplemented with 10% FCS (Sigma-Aldrich), 2 mM L-glutamine, 50 µM 2-mercaptoethanol, 100 µg/mL streptomycin and 100 U/mL penicillin (all Life Technologies) (R10 media) at 37 °C, 5% CO_2_. 293T, COS-7 and L929 cells were similarly passaged, in DMEM media (Life Technologies) supplemented with 10% FCS, 2 mM L-glutamine, 100 µg/mL streptomycin and 100 U/mL penicillin (D10 media).

### Plasmid Constructs and Transduction of B16.Kb^loss^ Cell Lines

B16-F1 and B16.Kb^loss^ cells were transduced, as described previously (30), with retroviral vectors containing a full-length membrane-bound form of HSV glycoprotein B (gB) and enhanced GFP (GFP) or CFP, respectively, to generate B16-F1-gB-GFP (B16.gB) and B16.Kb^loss^-gB-CFP (B16.Kb^loss^.gB) cell lines. Briefly, retroviruses were generated by transfecting the 293T cell lines with pMIG-gB or pMIC-gB, pMD.old.gag.pol, and pCAG-VSVG. B16 cells were next transduced with filtered retrovirus supernatant in the presence of 8 µg/mL polybrene (Sigma-Aldrich). For the generation of B16.Kb^loss^.gB_IFN cell lines for vaccination, murine IFNα1, IFNα2, IFNα4, IFNα5, IFNα6, IFNα9, and IFNβ was amplified from the pkCMVint mammalian expression vector (31) and subcloned into pMIG, as described previously (29). B16.Kb^loss^.gB cells were then retrovirally transduced with the pMIG-IFN constructs, and GFP^+^ cells sorted using a BD FACSAriaIII cell sorter (BD Biosciences) to select stable B16Kb^loss^.gB_IFN cell lines. GFP expression of sorted cell lines was confirmed using a BD LSRFortessa X-20 (BD Biosciences).

### Type I IFN Bioassay

Bioactive IFNα/β secretion was confirmed and quantitated using an *in vitro* IFN bioassay (32). Briefly, L929 cells were exposed to serial dilutions of acid-treated supernatants from the engineered B16.Kb^loss^.gB_IFN cell lines or NIH IFNα/β standard (1,000 IU/mL). Cell supernatants were collected from 5x10^5^ irradiated cells after 24 h in culture. After 24 h, encephalomyocarditis virus (EMCV) was added to each well. Following a further 24 h, end-point titers were defined as the dilution producing a 50% reduction in cytopathic effect (CPE) of the L929 cells. Bioactive titers were calculated by comparing the CPE of the B16 or COS-7 cell supernatants to the IFNα/β standard.

### Mice

C57BL/6 (B6) female mice that express the CD45.2 allele were purchased from the Animal Resources Centre, Murdoch, Western Australia. gB-specific T-cell receptor (TCR) transgenic (gBT.I) mice that express the CD45.1 allele (33), type I IFN knockout mice (IFNAR1^o/o^) (34), XCR1-DTRvenus mice (XCR1-DTR) (35), and I-A/E knockout mice (IA/E^o/o^) (36) were bred at the Telethon Kids Institute. Mice were typically used at 8-12 weeks. Animals were housed under pathogen-free conditions and all studies were approved by the Telethon Kids Institute’s Animal Ethics Committee (AEC) (AEC#290, AEC#295, AEC#325, AEC#348).

### Preparation of T Cells

For transfer of precursor gBT.I cells, single cell suspensions were prepared from pooled lymph nodes from naïve gBT.I female mice. Purity of gBT.I CD8^+^ T cells was determined by flow cytometry, and 5 x 10^4^ CD45.1^+^ Vα2^+^ CD8^+^ gBT.I T cells were washed and resuspended in 200 µL RPMI for i.v. injection into recipient mice at least one day prior to whole-cell vaccination.

### Whole-Cell Vaccination Strategy

Mice were vaccinated i.p. with 2.5 x 10^6^ irradiated (200 Gy) B16Kb^loss^.gB or B16Kb^loss^.gB_IFN cells. Cells were washed in PBS prior to irradiation and resuspended in 300 µL PBS for injection. For recombinant IFN experiments, mice received 2.5 x 10^6^ irradiated (200 Gy) B16Kb^loss^.gB cells at the same time as injection with 10^5^ IU IFNα1 or IFNβ, produced in-house as previously described (31, 37).

### Depletion/Blocking Experiments

For XCR1 depletion experiments, XCR1-DTR mice were administered with either PBS control or 25 ng/g weight diphtheria toxin (Sigma) one day prior to vaccination. For NK depletion experiments, mice received control PBS or 200 µg anti-NK1.1 (BioXCell) one day before and after vaccination. For CD40L blocking experiments, mice were administered with either control IgG isotype (BioXCell) or 200 µg anti-CD40L blocking antibody (BioXCell) on the same day as vaccination.

### Flow Cytometry

Spleen, lymph nodes and/or tumors were harvested and passed through a 70 µm metal mesh and red blood cell lysed. Resulting single cell suspensions were stained with monoclonal antibodies specific for mouse CD8α (53-6.7), CD45.1 (A20), Vα2 (B20.1), IFN*γ* (XMG1.2), PD-1 (29F-1412), MHCI (AF6-88.5), CD45 (30-F11), and/or NK1.1 (PK1136) (all from BD Biosciences). For *ex vivo* cytokine production assays, splenocytes were first restimulated with 1 µM gB_498-505_ peptide for 1 h at 37 °C prior to addition of 0.22 mg Brefeldin A (BD GolgiPlug™, BD Biosciences) for a further 4 h. Following surface stain, cells were fixed with 4% paraformaldehyde prior to permeabilization with Permeabilization Buffer (eBioscience) and staining with IFN*γ*. Cells were stained with Fixable Viability Stain 575V at 1:20,000 (BD Biosciences) prior to surface staining or propidium iodide (PI; Sigma) immediately prior to acquisition to exclude dead cells. Cells were analyzed using the BD LSRFortessa and FlowJo software (BD Biosciences/TreeStar).

### Tumor Challenge and Treatment

Mice were injected subcutaneously with 5 x 10^5^ B16 wildtype or B16.gB cells in 50 µL of RPMI media. Mice were then vaccinated, as described above, three days (for survival experiments) or four days (for T cell infiltration experiments) post-tumor inoculation. Mice receiving CPB were injected i.p. with 200 µg anti-PD-L1 (BioXCell) on days six, nine, and twelve post-tumor inoculation. Tumor size was monitored using calipers and tumor volume was calculated using the following formula: (length (mm) x width (mm)^2^)/2. Mice with tumors >1000 mm^3^ were euthanized. Tumor-free mice are defined as mice with no palpable masses.

### Statistics

All statistical analyses were performed using GraphPad (GraphPad Software Inc. v7.0a). Comparison of MFI, IFN titers, MHCI allele expression, and T cell expansion was assessed using one-way or two-way ANOVA. Difference in tumor survival was compared using the Log-Rank Mantel-Cox test. Statistical significance is indicated as *p < 0.05, **p < 0.01, ***p < 0.005, and ****p < 0.001, unless otherwise stated.

## Results

### Generation of B16 Cell Lines Expressing Glycoprotein B and Functional Type I IFN

**Figure 1:**
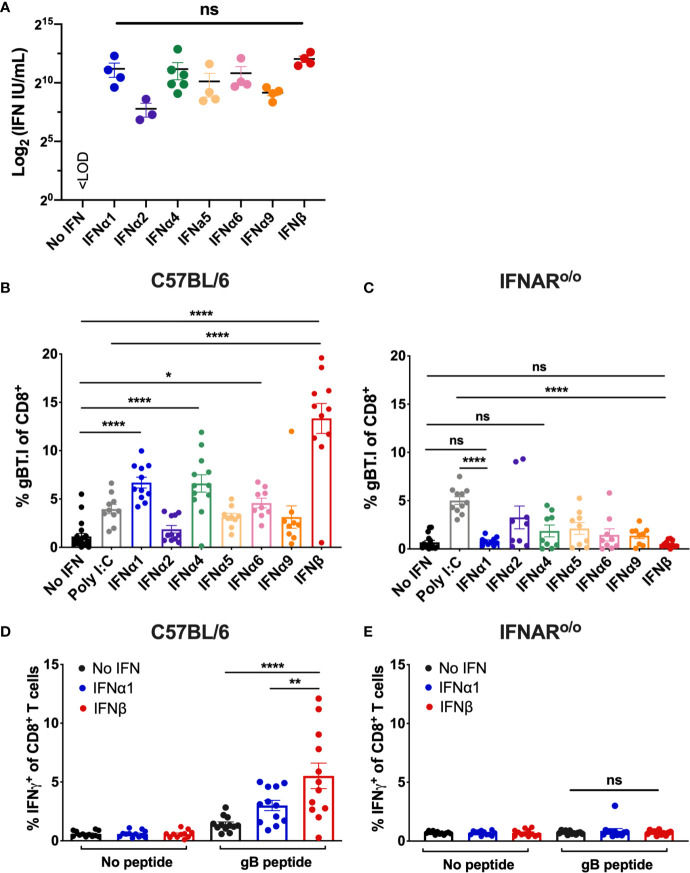
Vaccination with IFNβ-secreting B16 cell lines significantly enhances transgenic and endogenous gB-specific CD8^+^ T cell expansion. **(A)** IFN concentration (mean ± SEM) as determined by a cytopathic bioassay comparing supernatants from the engineered B16.Kb^loss^.gB_IFN cell lines with an IFNα/β standard (n = 3-6). **(B–E)** Wildtype C57BL/6 or IFNAR^o/o^ mice received 5 x 10^4^ naïve gBT.I cells one day prior to vaccination with 2.5 x 10^6^ irradiated B16.Kb^loss^.gB or B16.Kb^loss^.gB_IFN cells (n = 9-11 per group) with spleens harvested seven days post-vaccination. Expansion of gBT.I cells **(B, C)** or endogenous IFN*γ* ^+^ CD8^+^ T cells post-restimulation with gB peptide **(D, E)** was measured by flow cytometry (n = 11-12 per group). Data is pooled from 2-4 independent experiments and analysed by one-way ANOVA, ****p < 0.001, **p < 0.01, *p < 0.05, ns, not significant. Bars represent mean ± SEM.

To compare the adjuvant potential of different type I IFN subtypes, we established a whole-cell vaccination model to systematically interrogate the capacity of individual IFN subtypes to enhance CD8^+^ T cell priming. We engineered B16 melanoma cells deficient in the MHC class I alloantigen H-2K^b^ haplotype (B16.Kb^loss^) (38) to stably express a model tumor antigen (Herpes Simplex Virus (HSV) derived glycoprotein B (gB)). The resulting B16.Kb^loss^.gB cell line is defective in direct presentation of K^b^-restricted gB epitopes by tumor cells to the CD8^+^ T cell compartment, providing a model system to evaluate cross-priming by DCs. A panel of seven distinct type I IFN subtypes were stably expressed to produce a suite of cell lines for vaccination (B16.Kb^loss^.gB_IFNα1, B16.Kb^loss^.gB_IFNα2, B16.Kb^loss^.gB_IFNα4, B16.Kb^loss^.gB_IFNα5, B16.Kb^loss^.gB_IFNα6, B16.Kb^loss^.gB_IFNα9 and B16.Kb^loss^.gB_IFNβ; collectively referred to as B16.Kb^loss^.gB_IFN). Cross-priming in our model system can be tracked by measuring the expansion of adoptively-transferred T cell receptor (TCR) transgenic T cells specific for the HSV immunodominant gB_498-505_ peptide (39) (gBT.I cells) and/or IFN*γ* production by CD8^+^ T cells following restimulation with gB_498-505_ (33). We used a cytopathic protective effects (CPE) assay (32) to validate transgenic expression of type I IFN subtypes. Quantification of bioactive type I IFN titers in the cell supernatants confirmed IFN production was robust and not significantly different among the different subtypes (**Figure 1A**).

### Whole-Cell Vaccination With IFNβ Significantly Expands Transgenic and Endogenous Tumor-Specific CD8^+^ T Cells in an IFNAR-Dependent Manner

The capacity of type I IFN subtypes to expand tumor-specific CD8^+^ T cells was investigated in cohorts of C57BL/6 mice inoculated with a single irradiated B16.Kb^loss^.gB_IFN cell line as a whole-cell vaccine. To track gB-specific responses, 5 x 10^4^ naïve gBT.I CD8^+^ T cells expressing the congenic marker CD45.1 were adoptively transferred into the mice prior to vaccination. An optimal saturating dose of 2.5 x 10^6^ irradiated cells was selected in a prior experiment by titration of B16.Kb^loss^.gB_IFNα4 cells and comparison of gBT.I CD8^+^ T cell expansion (**Supplementary Figure 1A**). Mice vaccinated with irradiated B16.Kb^loss^.gB_IFN cells producing 4 out of the 7 IFN subtypes tested (IFNα1, IFNα4, IFNα6 or IFNβ) induced significantly greater expansion of gBT.I CD8^+^ T cells compared to vaccination with control irradiated B16.Kb^loss^.gB cells expressing no IFN (**Figure 1B**). Notably, mice receiving B16.Kb^loss^.gB_IFNβ cells showed the most striking increase in T cell expansion over and above all other subtypes tested, as well as vaccination with a commonly used adjuvant in the clinic (40), polyinosinic-polycytidylic acid (poly I:C). This trend was also observed when using recombinant doses of two of our highest-performing IFN subtypes, IFNα1 and IFNβ. Vaccination with irradiated B16.Kb^loss^.gB cells in combination with 1 x 10^5^ U IFNβ, but not IFNα1, produced significantly enhanced CD8^+^ T cell expansion compared to vaccination with the cell inoculum alone, indicating a subtype-intrinsic effect (**Supplementary Figure 1B**).

Next, we performed our whole-cell vaccination protocol in IFNAR^o/o^ mice that lack the receptor through which all type I IFNs signal (34). We first verified that transferred transgenic gBT.I cells were not rejected in IFNAR^o/o^ mice by confirming similar persistence 30 days post-transfer to that observed in wildtype C57BL/6 mice (**Supplementary Figure 1C**). Expansion of transferred naïve gBT.I CD8^+^ T cells in response to vaccination was abrogated in IFNAR^o/o^ mice, demonstrating a requirement for signaling through IFNAR on host cells for IFN to have adjuvant activity (**Figure 1C**). Interestingly, increased CD8^+^ T cell expansion in IFNAR^o/o^ mice was observed following vaccination with the B16.Kb^loss^.gB line in combination with the adjuvant poly I:C, suggesting IFNAR-independent mechanisms for this adjuvant.

We next asked if enhanced recruitment of gB-specific CD8^+^ T cells during vaccination also occurred in the endogenous T cell compartment, selecting two of our strongest performing adjuvant candidates, IFNα1 and IFNβ, for the remainder of our analyses. Splenocytes from vaccinated C57BL/6 mice were re-stimulated *ex vivo* with the immunodominant gB_498-505_ peptide (41) and the percentage of IFN*γ* ^+^ CD8^+^ T cells was measured as a marker of vaccine-specific CD8^+^ T cell expansion. Consistent with our results from transgenic T cell experiments, endogenous gB-specific CD8^+^ T cell expansion was significantly enhanced by vaccination with IFNβ compared to IFNα1 or no adjuvant (1.84- and 3.89-fold increase, p=0.004 and p<0.0001 respectively; **Figure 1D**), which was abrogated in IFNAR^o/o^ mice (**Figure 1E**). A sustained increase in gB-specific CD8^+^ T cells was observed 60 days post-vaccination, suggesting the potential for long-lived anti-tumor responses (**Supplementary Figure 2**).

### IFNβ-Mediated Expansion of Tumor-Specific CD8^+^ T Cells Is Dependent on XCR1^+^ DCs, CD4^+^ T Cells and CD40-CD40L Signaling

**Figure 2:**
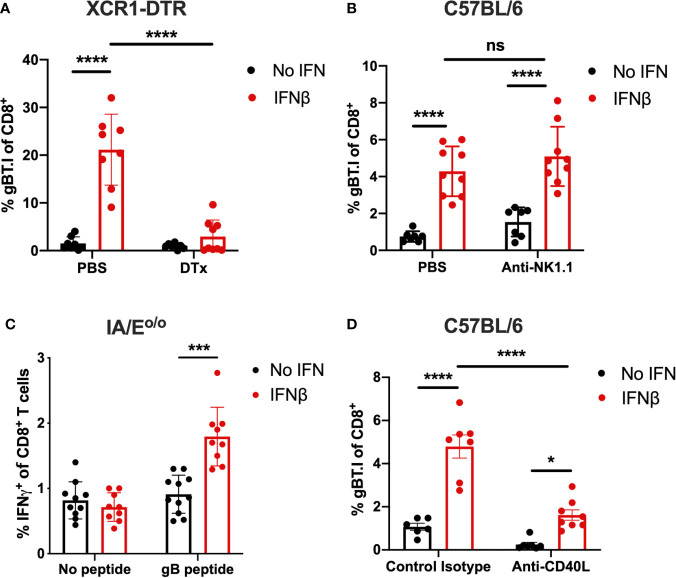
IFNβ-mediated expansion of gB-specific CD8^+^ T cells is dependent on XCR1^+^ DCs, CD4^+^ T cells and CD40/CD40L signalling. Expansion of 5 x 10^4^ transferred naïve gBT.I **(A, B, D)** or endogenous gB-specific CD8^+^ T cells **(C)** was measured seven days post-vaccination with 2.5 x 10^6^ irradiated B16.Kb^loss^.gB ± IFNβ cells. **(A)** XCR1-DTR mice received either PBS control or 25 ng/g weight diphtheria toxin (DTx) one day prior to vaccination to deplete XCR1^+^ DCs (n = 8 per group). **(B)** C57BL/6 mice received control PBS or 200 µg anti-NK1.1 one day prior and post vaccination (n = 7-9 per group). **(C)** Splenocytes from IFNβ-vaccinated IA/E^o/o^ mice were restimulated with gB peptide and IFN*γ* ^+^ endogenous CD8^+^ T cells were measured (n = 9-11 per group). **(D)** C57BL/6 mice received control isotype or 200 µg anti-CD40L on the same day as vaccination (n = 6-8 per group). Data is pooled from two independent repeats and analysed by one-way **(C)** or two-way **(A, B, D)** ANOVA, ****p < 0.001, ***p < 0.005, *p < 0.05, ns, not significant. Bars represent mean ± SEM.

Considering IFN does not directly act on CD8^+^ T cells to drive T cell expansion, as demonstrated by the lack of expansion of transferred gBT.I cells in IFNAR^o/o^ mice, we next focused on the role of specific cell types that may be critical for driving enhanced CD8^+^ T cell expansion. Given that the tumor cells comprising the vaccine inoculum are unable to present the K^b^-restricted gB antigen directly to CD8^+^ T cells, we evaluated cross-priming by professional antigen-presenting cells. We and others (30, 42–44) have demonstrated previously that XCR1^+^ cross-presenting DCs are the key cell type cross-priming anti-tumor CD8^+^ T cell immunity. To determine whether these cells were responsible for the cross-priming in our vaccination model, we utilized XCR1-DTR mice (35) to selectively deplete XCR1^+^ DCs immediately prior to vaccination with irradiated B16.Kb^loss^.gB_ IFNβ cells. Consistent with the critical role XCR1^+^ DCs are reported to play in cross-priming, the enhanced gBT.I expansion observed post-vaccination with IFNβ in control mice was completely abrogated in the XCR1^+^ DC-depleted mice, confirming that these cells are essential for priming the observed CD8^+^ T cell response in this setting (**Figure 2A**).

NK/DC signaling can occur *via* Flt3L (45), IFN*γ* and TNFα (12) and may be a crucial factor in augmenting cross-priming and anti-tumor responses (46–48). Therefore, we hypothesized that NK cells may also contribute to the enhanced cross-priming mediated by IFNβ in our model. To assess this, endogenous NK cells were depleted one day before and after vaccination with irradiated B16.Kb^loss^.gB_IFNβ cells (**Supplementary Figures 3A,B**). However, no effect on the expansion of transferred tumor-specific transgenic CD8^+^ T cells was observed in these mice (**Figure 2B**). We next considered the role of CD4^+^ T cells in our model, which can license DCs for successful cross-priming (49). Helper T cell dependence was indicated by the poor induction of endogenous IFN*γ*-producing gB-specific CD8^+^ T cells in MHC class II-deficient mice (I/AE^o/o^) (**Figure 2C**). Whilst significant expansion of tumor-specific T cells remained following vaccination with IFNβ in I/AE^o/o^ mice, a greater than 3-fold decrease (5.5% vs 1.8%, p < 0.0005) was observed when compared to the percentage of IFN*γ* ^+^ CD8^+^ T cells in wildtype C57BL/6 mice (**Figure 1D**). We hypothesized that the observed T-helper dependence could reflect a requirement for CD40/CD40L signaling. To this end, we blocked CD40L in mice vaccinated with IFNβ and measured the expansion of transferred gBT.I CD8^+^ T cells. There was approximately a 3-fold decrease in expansion between control isotype- and anti-CD40L-treated mice following whole-cell vaccination with IFNβ (4.8% vs 1.6%, p < 0.0001) (**Figure 2D**), suggesting a dependence on CD40L signaling for optimal CD8^+^ T cell priming.

### Vaccination With IFNβ Increases Tumor-Specific CD8^+^ T Cell Infiltration and Delays Tumor Progression

**Figure 3:**
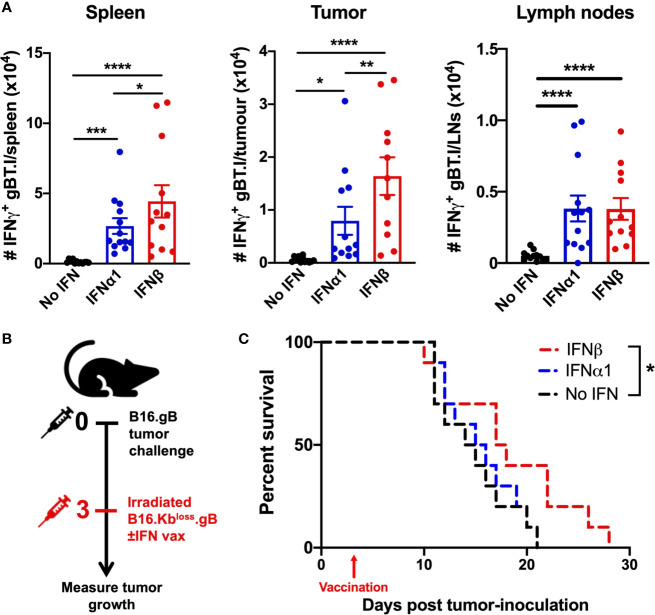
Increased tumor-reactive CD8^+^ T cell infiltration into the tumor and prolonged survival in mice vaccinated with IFNβ-secreting B16 cell lines. **(A)** B16.gB tumor-bearing mice received 5 x 10^4^ naïve gBT.I cells one day prior to vaccination with 2.5 x 10^6^ irradiated B16.Kb^loss^.gB ± IFN cells. Seven days post-vaccination, expansion of IFN*γ*-producing gBT.I, in response to restimulation with gB peptide was enumerated in the spleen, tumor, and ipsilateral lymph nodes (axillary, brachial and inguinal) (n = 12 per group). **(B, C)** Experiment schematic **(B)** and survival curves **(C)** of mice receiving vaccination with 2.5 x 10^6^ irradiated B16.Kb^loss^.gB ± IFN cells (n = 10 per group). Data is pooled from 2-3 biologically independent repeats and analysed by one-way ANOVA for **(A)** and Log-rank Mantel-Cox test for **(C)**, ****p < 0.001, ***p < 0.005, **p < 0.01, *p < 0.05. Bars represent mean ± SEM.

We next investigated the impact of whole-cell vaccination with IFNβ on circulating T cells and infiltration into the tumor microenvironment (TME) in mice bearing B16.gB tumors. Re-stimulation of lymphocytes *ex vivo* with gB_498-505_ peptide demonstrated an increase in the number of endogenous gB-specific CD8^+^ T cells in the spleen, tumor and lymph nodes of mice vaccinated with IFNα1 or IFNβ compared to vaccination alone (**Figure 3A**). Furthermore, IFNβ-vaccinated mice showed significantly higher numbers of tumor-specific CD8^+^ T cells in the spleen and tumor relative to IFNα1-vaccinated mice suggesting successful tumor-infiltration of functional, tumor-reactive CD8^+^ T cells.

We next sought to determine the therapeutic potential of our vaccination strategy. Vaccination three days post-B16.gB tumor inoculation resulted in a significant increase in survival for the IFNβ-vaccinated cohort (18.4 ± 6.1 days) relative to vaccination with B16.Kb^loss^.gB cells without IFN (14.8 ± 3.7 days, p < 0.0323) (**Figures 3B, C** and **Supplementary Figure 4**). Strikingly, 40% of mice in the IFNβ-vaccinated group survived to day 21, compared to 0% of mice receiving vaccination alone or with IFNα1. Therefore, whilst both IFNα1- and IFNβ-vaccination has the capacity to increase tumor-specific T cell infiltration into the TME, only IFNβ resulted in a therapeutic benefit.

### Anti-PD-L1 CPB Combined With Vaccination Plus IFNβ Promotes Overall Survival

**Figure 4:**
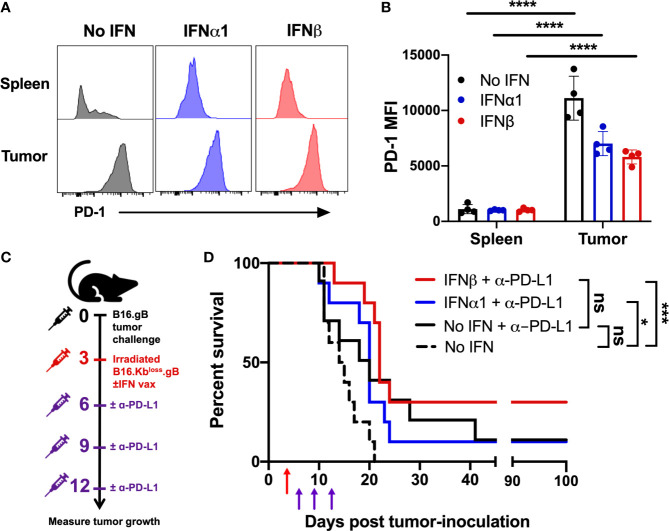
Vaccination with IFNβ improves overall survival in combination with anti-PD-L1 checkpoint blockade therapy. Mice were vaccinated with 2.5 x 10^6^ irradiated B16.Kb^loss^.gB ± IFN cells three days post-B16.gB tumor inoculation. **(A, B)** Representative histograms **(A)** and mean fluorescence intensity (MFI) **(B)** of PD-1 expression on transferred transgenic CD8^+^ T cells (gBT.I) from mice harvested seven days post-vaccination (n = 4 per group). **(C, D)** Experiment schematic **(C)** and survival curves **(D)** of mice receiving vaccination ± three doses of anti-PDL1 treatment (200 µg/dose) on days six, nine and twelve post-tumor challenge (n = 10 per group from two independent experiments). Dashed black line indicates the no IFN group from the vaccination alone cohorts in Fig 3c, as these data belong to the same experiment but were separated for clarity. Data was analysed by two-way ANOVA, comparing tumor to spleen **(B)** or Log-rank Mantel-Cox test **(D)**, ****p < 0.001 ***p < 0.005, *p < 0.05, ns, not significant.

It is well established that the immunosuppressive TME can induce upregulation of inhibitory markers on infiltrating immune cells, which can be overcome by CPB (50). For example, infiltration of the tumor by T cells expressing the inhibitory receptor PD-1 is associated with response to anti-PD-1 therapy (51). We assessed PD-1 expression on tumor-infiltrating transgenic CD8^+^ T cells seven days post-vaccination and observed a significant upregulation across all groups relative to gBT.I T cells in the spleen (**Figures 4A, B**). These data provided a rationale to investigate whether vaccination with IFNβ would sensitize mice to anti-PD-L1 CPB. Mice were vaccinated three days post-tumor inoculation and dosed with anti-PD-L1 on days three, six, and nine post-vaccination (**Figure 4C**). Both IFNα1- and IFNβ-vaccination, but not vaccination without IFN (no IFN), significantly increased survival when used in combination with anti-PD-L1 relative to vaccination alone (**Figure 4D** and **Supplementary Figure 5**). When compared to treatment with anti-PD-L1 in the absence of IFN, the combination of IFNβ-vaccination and anti-PD-L1 appeared to further increase overall survival, which however, did not reach a statistical significance. Notably, 30% of mice receiving IFNβ-vaccination plus anti-PD-L1 displayed complete tumor regression, surviving to at least 100 days post-tumor inoculation. In contrast, only 10% of anti-PD-L1 treated mice displayed this complete regression in the IFNα1-vaccination or vaccine alone groups. Thus, vaccination strategies incorporating IFNβ might function in conjunction with anti-PD-L1 treatment to promote overall survival. Interestingly, when comparing tumors harvested at endpoint from mice treated with or without anti-PD-L1, anti-PD-L1 appears to promote gB antigen downregulation (p=0.0079; **Supplementary Figures 6A, B**). Surviving mice were re-challenged with wildtype B16 tumors, with a significant increase in survival observed in mice initially receiving IFNβ-vaccination relative to naïve, vaccine-alone or IFNα1-vaccinated mice (**Supplementary Figures 6C, D**). Taken together, these data suggest that enhanced epitope spreading may occur during IFNβ-vaccination.

## Discussion

Here we report a systematic approach to determine the adjuvant potential of distinct type I IFN subtypes in a whole-cell cancer vaccine model. We provide compelling evidence that several type I IFN subtypes can significantly enhance cross-priming of tumor-specific CD8^+^ T cells when compared to no adjuvant or a gold standard adjuvant, poly I:C. Critically, not all type I IFNs possess this capacity. When testing our two highest-performing subtypes therapeutically, we observe that vaccination with IFNβ was superior at enhancing survival in a preclinical model of melanoma compared to vaccination alone. In addition, therapeutic vaccination with IFNβ delayed tumor progression and could be administered in combination with immune CPB to favor overall survival. Collectively, these results highlight that IFNβ is a potent adjuvant for cancer vaccination strategies.

Immune adjuvants are key components of cancer vaccines, providing obligatory danger signals for DC-licensing and the promotion of efficient immune responses (52). For example, the commonly used adjuvant poly I:C is a toll-like receptor 3 ligand that can induce type I IFN expression (53) and enhance the cross-priming of tumor-associated antigen (54–56). The renewed interest in cancer vaccination follows advances in neoantigen discovery and the development of CPB. Novel adjuvants that enhance the priming of tumor-reactive T cells will synergize with these approaches and improve clinical outcomes (57). Type I IFN has previously been demonstrated to be a prime candidate due to its ability to promote cross-priming in both a viral (18) and tumor (19–21, 23, 58) context. Whilst others have highlighted the ability of specific subtypes (16, 23) to enhance cross-priming, this is the first study to directly compare multiple type I IFN subtypes head-to-head. We identified IFNβ as a superior adjuvant, demonstrating an enhanced capacity to expand CD8^+^ T cells post-vaccination. These T cells were shown to be both functional and capable of infiltrating the TME. Notably, the observed differences between type I IFN subtypes in our model were subtype-intrinsic, with similar doses of recombinant IFNα1 and IFNβ eliciting differing magnitudes of T cell expansion. These experiments were critical given the difficulty in definitively quantitating IFN production in engineered cell lines with currently available assays (29). To enhance studies in this area, there is undoubtedly a need for improved tools to be developed allowing accurate measurement of both mouse and human individual type I IFN subtypes. Whilst we observed enhanced cross-priming by a number of type I IFN subtypes when compared to the established vaccine adjuvant, poly I:C, we identified that poly I:C was acting in an IFNAR-independent manner in our model. Consistent with this, it has previously been reported that poly I:C stimulation of DCs from IFNAR^o/o^ mice induces 354 differentially expressed genes (DEG) as compared to 988 DEG induced in DCs from wildtype mice, demonstrating the presence of IFNAR-independent mechanisms (59). Clearly, further studies are warranted to dissect the mechanisms underlying the adjuvant activities of poly I:C.

We have previously shown that distinct IFNα subtypes display diverse anti-cancer activities, noting that IFNα paralogs clustered together on individual chromosomes behave similarly (29). Previous studies indicate differences in biological activity between the human type I IFN subtypes (28) due to a variety of mechanisms including variation in binding affinity for the IFNAR subunits (60, 61); stability of the IFN/IFNAR complex (62); and sensitivity to negative feedback causing desensitization to IFN signaling (63). Accordingly, IFNβ appears to provoke a more sustained IFN signaling than its IFNα counterparts (63), and induces a unique gene expression program (61), which may collectively underly the superior ability of IFNβ to promote cross-priming observed in our study. Further studies are required to determine if these molecular mechanisms underpin this observation. Whilst the direct human orthologs of the murine IFNα subtypes are unknown, the presence of a single, distinct IFNβ subtype in both species raises the possibility that human IFNβ may similarly enhance vaccination protocols as we found in our murine model. Taken together with these observations, our data provides critical proof-of-concept for the investigation into the adjuvant efficacies of human type I IFN subtypes.

The pleiotropic nature of type I IFNs prompted our investigation into mechanisms underlying enhanced cross-priming. Type I IFNs can act on CD8α^+^ DCs to promote maturation and cross-priming (17–21). These DCs broadly comprise the cross-presenting DC subset (specifically the XCR1^+^ DC population (42, 43, 64)) and have been shown to be crucial in mediating effective anti-tumor responses (65). Here we establish that cross-presenting XCR1^+^ DCs are essential for enhanced priming of tumor-specific CD8^+^ T cells during vaccination with IFNβ. Further studies are required to determine if IFNβ is directly acting on these cross-presenting DCs. For successful cross-presentation by XCR1^+^ DCs, licensing is critical. DC licensing typically occurs through CD40L engagement by helper CD4^+^ T cells, but can also occur by stimulation with soluble factors, such as those produced by NK cells (49). It has previously been proposed that type I IFN abrogates the requirement of CD4^+^ help for successful cross-priming (16, 18, 66, 67). Contrary to this proposition, we have identified that optimal cross-priming post-vaccination with IFNβ was CD4^+^ T cell-dependent and required CD40/CD40L signaling for effective CD8^+^ T cell expansion. It is possible that the reported CD4^+^ T cell-independent responses could reflect a context-dependent requirement for CD40/CD40L signaling or alternatively be driven by specific type I IFN subtypes. For the optimal development of vaccine strategies incorporating specific type I IFNs in humans, a clear understanding of the helper requirement status is essential.

Our data demonstrates the vital role adjuvants play in mediating vaccine responses, with the incorporation of IFNβ boosting T cell expansion and delaying tumor progression. One of the major goals of cancer vaccination is to expand a population of tumor-specific T cells, and as such it is a prime candidate to overlay with CPB to improve clinical outcomes (57). Indeed, the upregulation of PD-1 we observed on tumor-infiltrating T cells highlights the therapeutic potential of combining these two treatment strategies. As a corollary, vaccination with IFNβ in our model was used in conjunction with anti-PD-L1 blockade to further enhance overall survival. Clearly there is an opportunity for vaccination to provide benefit to patients predicted to fail to respond to CPB, by stimulating tumor-reactive T cell responses in those with insufficient T cell infiltrate. While there have been significant advances in the field of neoantigen discovery for the development of personalized cancer vaccination strategies (68), there is clearly scope to improve adjuvants that optimally harness next-generation vaccines (57) and improve unsatisfactory response rates to cancer vaccination currently observed in the clinic (2, 52). Here, we propose IFNβ as a novel adjuvant candidate. As new synthetic cancer vaccines become available, we speculate that overlaying these new strategies with IFNβ could enhance response rates, both alone and in combination with CPB.

Whilst we observed a striking 30% survival rate with IFNβ vaccination and CPB in mice bearing highly aggressive B16 melanoma tumors, the majority developed progressive disease. The loss of gB expression observed in the tumors of non-responding IFNβ-vaccinated mice, and the eventual tumor outgrowth observed despite persistent gB-specific T cell responses, collectively indicate antigen loss to be a likely contributing factor of tumor escape in our model. A major downfall of current immunotherapy strategies is the likelihood for recurrence in those patients that initially respond (69). Combating escape mechanisms, such as antigen loss, will be crucial in the generation of long-lasting effective treatments for cancer patients (70). An improved cancer vaccine incorporating IFNβ as a highly potent adjuvant may also limit the chance for tumor escape. Indeed, the delayed tumor progression observed in IFNβ-vaccinated survivors re-challenged with wildtype tumors, whilst limited by the number of surviving mice available, draws us to speculate that IFNβ could similarly be a candidate to mediate the immune phenomenon of epitope spreading. Treatments that simultaneously promote on-target anti-tumor responses whilst generating novel immune responses to a broader spectrum of antigens should be considered a priority.

In summary, our data establishes that distinct type I IFN subtypes elicit potent anti-tumor immune responses through cross-priming of tumor-reactive T cells, highlighting their untapped anti-cancer potential. Notably, we identified IFNβ as a superior adjuvant, providing clear rationale for its incorporation into future cancer vaccine protocols. Since the approval of IFNα2 for use against advanced melanoma over 30 years ago (71), the therapeutic potential of other subtypes has been largely understudied. The remarkable superiority of IFNβ in our study, and evidence that not all IFNα subtypes are equal, advocates for the re-evaluation of human type I IFN subtypes used clinically to maximize their clinical efficacy as potent immunomodulators.

## Data Availability Statement

The raw data supporting the conclusions of this article will be made available by the authors, without undue reservation.

## Ethics Statement

The animal study was reviewed and approved by Telethon Kids Institute Animal Ethics Committee.

## Author Contributions

KA, TW, BF, and JW designed the experiments. KA, TW, CT, HN, ACB and SB performed the experiments and analyzed the data. KA, BF and JW wrote the manuscript. K.A, TW, HN, SB, BW, JA, AB, AM, MC, BF and JW edited the manuscript. AB, AM, VF, BF and JW provided supervision. All authors contributed to the article and approved the submitted version.

## Funding

This work was supported by the Richard Walter Gibbon Medical Research Scholarship and Rachel Kierath Top-Up Scholarship in Paediatric Cancer Research (scholarships to KA), Australian Government Research Training Program Scholarship at The University of Western Australia (scholarships to HN, ACB, and SB), the Cancer Council Western Australia (fellowships to AM and JW), and grants from the Brady Cancer Support Foundation and host institute.

## Conflict of Interest

The authors declare that the research was conducted in the absence of any commercial or financial relationships that could be construed as a potential conflict of interest.

## Publisher’s Note

All claims expressed in this article are solely those of the authors and do not necessarily represent those of their affiliated organizations, or those of the publisher, the editors and the reviewers. Any product that may be evaluated in this article, or claim that may be made by its manufacturer, is not guaranteed or endorsed by the publisher.

## References

[1] Ott PA Hu Z Keskin DB Shukla SA Sun J Bozym DJ. An Immunogenic Personal Neoantigen Vaccine for Patients With Melanoma. Nature (2017) 547:217–21. 10.1038/nature22991 PMC5577644 28678778

[2] Hu Z Ott PA Wu CJ. Towards Personalized, Tumour-Specific, Therapeutic Vaccines for Cancer. Nat Rev Immunol (2018) 18:168–82. 10.1038/nri.2017.131 PMC6508552 29226910

[3] Zhang Y Ma J-A Zhang H-X Jiang Y-N Luo W-H. Cancer Vaccines: Targeting KRAS-Driven Cancers. Expert Rev Vaccines (2020) 19:163–73. 10.1080/14760584.2020.1733420 32174221

[4] Sahin U Derhovanessian E Miller M Kloke BP Simon P Löwer M. Personalized RNA Mutanome Vaccines Mobilize Poly-Specific Therapeutic Immunity Against Cancer. Nature (2017) 547:222–6. 10.1038/nature23003 28678784

[5] Isaacs A Lindenmann J Andrewes CH. Virus interference. I. The Interferon. Proceedings of the Royal Society of London. Ser B - Biol Sci (1957) 147:258–67. 10.1098/rspb.1957.0048 13465720

[6] Gresser ION Bourali C. Exogenous Interferon and Inducers of Interferon in the Treatment of Balb/c Mice Inoculated With RC 19 Tumour Cells. Nature (1969) 223:844–5. 10.1038/223844a0 5799031

[7] Bart RS Porzio NR Kopf AW Vilcek JT Cheng EH Farcet Y. Inhibition of Growth of B16 Murine Malignant Melanoma by Exogenous Interferon. Cancer Res (1980) 40:614–9. 6162552

[8] Gato-Cañas M Zuazo M Arasanz H Ibañez-Vea M Lorenzo L Fernandez-Hinojal G. PDL1 Signals Through Conserved Sequence Motifs to Overcome Interferon-Mediated Cytotoxicity. Cell Rep (2017) 20:1818–29. 10.1016/j.celrep.2017.07.075 28834746

[9] Hervas-Stubbs S Perez-Gracia JL Rouzaut A Sanmamed MF Le Bon A Melero I. Direct Effects of Type I Interferons on Cells of the Immune System. Clin Cancer Res (2011) 17:2619–27. 10.1158/1078-0432.CCR-10-1114 21372217

[10] Zitvogel L Galluzzi L Kepp O Smyth MJ Kroemer G. Type I Interferons in Anticancer Immunity. Nat Rev Immunol (2015) 15:405–14. 10.1038/nri3845 26027717

[11] Salazar-Mather TP Ishikawa R Biron CA. NK Cell Trafficking and Cytokine Expression in Splenic Compartments After IFN Induction and Viral Infection. J Immunol (1996) 157:3054–64. 8816415

[12] Biron CA Nguyen KB Pien GC Cousens LP Salazar-Mather TP. Natural Killer Cells in Antiviral Defense: Function and Regulation by Innate Cytokines. Annu Rev Immunol (1999) 17:189–220. 10.1146/annurev.immunol.17.1.189 10358757

[13] Marrack P Kappler J Mitchell T. Type I Interferons Keep Activated T Cells Alive. J Exp Med (1999) 189:521–30. 10.1084/jem.189.3.521 PMC2192920 9927514

[14] Kolumam GA Thomas S Thompson LJ Sprent J Murali-Krishna K. Type I Interferons Act Directly on CD8 T Cells to Allow Clonal Expansion and Memory Formation in Response to Viral Infection. J Exp Med (2005) 202:637–50. 10.1084/jem.20050821 PMC2212878 16129706

[15] Braun D Caramalho I Demengeot J. IFN-α/β Enhances BCR-Dependent B Cell Responses. Int Immunol (2002) 14:411–9. 10.1093/intimm/14.4.411 11934877

[16] Le Bon A Etchart N Rossmann C Ashton M Hou S Gewert D. Cross-Priming of CD8^+^ T Cells Stimulated by Virus-Induced Type I Interferon. Nat Immunol (2003) 4:1009–15. 10.1038/ni978 14502286

[17] Dunn GP Bruce AT Sheehan KC Shankaran V Uppaluri R Bui JD. A Critical Function for Type I Interferons in Cancer Immunoediting. Nat Immunol (2005) 6:722–9. 10.1038/ni1213 15951814

[18] Lapenta C Santini SM Spada M Donati S Urbani F Accapezzato D. IFN-Alpha-Conditioned Dendritic Cells are Highly Efficient in Inducing Cross-Priming CD8^+^ T Cells Against Exogenous Viral Antigens. Eur J Immunol (2006) 36:2046–60. 10.1002/eji.200535579 16856207

[19] Diamond MS Kinder M Matsushita H Mashayekhi M Dunn GP Archambault JM. Type I Interferon is Selectively Required by Dendritic Cells for Immune Rejection of Tumors. J Exp Med (2011) 208:1989–2003. 10.1084/jem.20101158 21930769 PMC3182061

[20] Fuertes MB Kacha AK Kline J Woo S-R Kranz DM Murphy KM. Host Type I IFN Signals are Required for Antitumor CD8^+^ T Cell Responses Through CD8α^+^ Dendritic Cells. J Exp Med (2011) 208:2005–16. 10.1084/jem.20101159 PMC3182064 21930765

[21] Lorenzi S Mattei F Sistigu A Bracci L Spadaro F Sanchez M. Type I IFNs Control Antigen Retention and Survival of CD8α^+^ Dendritic Cells After Uptake of Tumor Apoptotic Cells Leading to Cross-Priming. J Immunol (2011) 186:5142–50. 10.4049/jimmunol.1004163 21441457

[22] Le Bon A Tough DF. Type I Interferon as a Stimulus for Cross-Priming. Cytokine Growth Factor Rev (2008) 19:33–40. 10.1016/j.cytogfr.2007.10.007 18068417

[23] Yang X Zhang X Fu ML Weichselbaum RR Gajewski TF Guo Y. Targeting the Tumor Microenvironment With Interferon-β Bridges Innate and Adaptive Immune Responses. Cancer Cell (2014) 25:37–48. 10.1016/j.ccr.2013.12.004 24434209 PMC3927846

[24] Hardy MP Owczarek CM Jermiin LS Ejdebäck M Hertzog PJ. Characterization of the Type I Interferon Locus and Identification of Novel Genes. Genomics (2004) 84:331–45. 10.1016/j.ygeno.2004.03.003 15233997

[25] Ives NJ Suciu S Eggermont AMM Kirkwood J Lorigan P Markovic SN. Adjuvant Interferon-α for the Treatment of High-Risk Melanoma: An Individual Patient Data Meta-Analysis. Eur J Cancer (2017) 82:171–83. 10.1016/j.ejca.2017.06.006 28692949

[26] Lavender KJ Gibbert K Peterson KE Van Dis E Francois S Woods T. Interferon Alpha Subtype-Specific Suppression of HIV-1 Infection *In Vivo* . J Virol (2016) 90:6001–13. 10.1128/JVI.00451-16 PMC4907223 27099312

[27] Brierley MM Fish EN. Review: IFN-Alpha/Beta Receptor Interactions to Biologic Outcomes: Understanding the Circuitry. J Interferon Cytokine Res (2002) 22:835–45. 10.1089/107999002760274845 12396722

[28] Gibbert K Schlaak JF Yang D Dittmer U. IFN-α Subtypes: Distinct Biological Activities in Anti-Viral Therapy. Br J Pharmacol (2013) 168:1048–58. 10.1111/bph.12010 PMC3594665 23072338

[29] Buzzai AC Wagner T Audsley KM Newnes HV Barrett LW Barnes S. Diverse Anti-Tumor Immune Potential Driven by Individual Ifnα Subtypes. Front Immunol (2020) 11:542. 10.3389/fimmu.2020.00542 32308653 PMC7145903

[30] Wylie B Seppanen E Xiao K Zemek R Zanker D Prato S. Cross-Presentation of Cutaneous Melanoma Antigen by Migratory XCR1^+^CD103- and XCR1^+^CD103^+^ Dendritic Cells. Oncoimmunology (2015) 4:e1019198. 10.1080/2162402X.2015.1019198 26405572 PMC4570138

[31] Cull VS Bartlett EJ James CM. Type I Interferon Gene Therapy Protects Against Cytomegalovirus-Induced Myocarditis. Immunology (2002) 106:428–37. 10.1046/j.1365-2567.2002.01423.x PMC1782722 12100732

[32] Seeds RE Miller JL. Measurement of Type I Interferon Production. Curr Protoc Immunol (2011) 92:14.21.1–14.21.11. 10.1002/0471142735.im1421s92 21400681

[33] Mueller SN Heath W McLain JD Carbone FR Jones CM. Characterization of Two TCR Transgenic Mouse Lines Specific for Herpes Simplex Virus. Immunol Cell Biol (2002) 80:156–63. 10.1046/j.1440-1711.2002.01071.x 11940116

[34] Müller U Steinhoff U Reis LF Hemmi S Pavlovic J Zinkernagel RM. Functional Role of Type I and Type II Interferons in Antiviral Defense. Science (1994) 264:1918–21. 10.1126/science.8009221 8009221

[35] Yamazaki C Sugiyama M Ohta T Hemmi H Hamada E Sasaki I. Critical Roles of a Dendritic Cell Subset Expressing a Chemokine Receptor, XCR1. J Immunol (2013) 190:6071–82. 10.4049/jimmunol.1202798 23670193

[36] Madsen L Labrecque N Engberg J Dierich A Svejgaard A Benoist C. Mice Lacking All Conventional MHC Class II Genes. Proc Natl Acad Sci USA (1999) 96:10338–43. 10.1073/pnas.96.18.10338 PMC17889 10468609

[37] Swaminathan N Lai CM Beilharz MW Boyer SJ Klinken SP. Biological Activities of Recombinant Murine Interferons Alpha 1 and Alpha 4: Large Difference in Antiproliferative Effect. Antiviral Res (1992) 19:149–59. 10.1016/0166-3542(92)90074-F 1332601

[38] Cho HI Lee YR Celis E. Interferon γ Limits the Effectiveness of Melanoma Peptide Vaccines. Blood (2011) 117:135–44. 10.1182/blood-2010-08-298117 PMC3037740 20889921

[39] Cose SC Kelly JM Carbone FR. Characterization of Diverse Primary Herpes Simplex Virus Type 1 gB-Specific Cytotoxic T-Cell Response Showing a Preferential V Beta Bias. J Virol (1995) 69:5849–52. 10.1128/jvi.69.9.5849-5852.1995 PMC189458 7543591

[40] Martins KAO Bavari S Salazar AM. Vaccine Adjuvant Uses of Poly-IC and Derivatives. Expert Rev Vaccines (2015) 14:447–59. 10.1586/14760584.2015.966085 25308798

[41] Wallace ME Keating R Heath WR Carbone FR. The Cytotoxic T-Cell Response to Herpes Simplex Virus Type 1 Infection of C57BL/6 Mice Is Almost Entirely Directed Against a Single Immunodominant Determinant. J Virol (1999) 73:7619. 10.1128/JVI.73.9.7619-7626.1999 10438852 PMC104289

[42] Dorner BG Dorner MB Zhou X Opitz C Mora A Güttler S. Selective Expression of the Chemokine Receptor XCR1 on Cross-Presenting Dendritic Cells Determines Cooperation With CD8^+^ T Cells. Immunity (2009) 31:823–33. 10.1016/j.immuni.2009.08.027 19913446

[43] Bachem A Hartung E Güttler S Mora A Zhou X Hegemann A. Expression of XCR1 Characterizes the Batf3-Dependent Lineage of Dendritic Cells Capable of Antigen Cross-Presentation. Front Immunol (2012) 3:214. 10.3389/fimmu.2012.00214 22826713 PMC3399095

[44] Broz ML Binnewies M Boldajipour B Nelson AE Pollack JL Erle DJ. Dissecting the Tumor Myeloid Compartment Reveals Rare Activating Antigen-Presenting Cells Critical for T Cell Immunity. Cancer Cell (2014) 26:638–52. 10.1016/j.ccell.2014.09.007 PMC4254577 25446897

[45] Barry KC Hsu J Broz ML Cueto FJ Binnewies M Combes AJ. A Natural Killer-Dendritic Cell Axis Defines Checkpoint Therapy-Responsive Tumor Microenvironments. Nat Med (2018) 24:1178–91. 10.1038/s41591-018-0085-8 PMC6475503 29942093

[46] Ferlazzo G Morandi B. Cross-Talks Between Natural Killer Cells and Distinct Subsets of Dendritic Cells. Front Immunol (2014) 5:159. 10.3389/fimmu.2014.00159 24782864 PMC3989561

[47] Müller L Aigner P Stoiber D. Type I Interferons and Natural Killer Cell Regulation in Cancer. Front Immunol (2017) 8:304. 10.3389/fimmu.2017.00304 28408907 PMC5374157

[48] Gerosa F Baldani-Guerra B Nisii C Marchesini V Carra G Trinchieri G. Reciprocal Activating Interaction Between Natural Killer Cells and Dendritic Cells. J Exp Med (2002) 195:327–33. 10.1084/jem.20010938 PMC2193595 11828007

[49] Heath WR Belz GT Behrens GM Smith CM Forehan SP Parish IA. Cross-Presentation, Dendritic Cell Subsets, and the Generation of Immunity to Cellular Antigens. Immunol Rev (2004) 199:9–26. 10.1111/j.0105-2896.2004.00142.x 15233723

[50] Sharma P Allison JP. Dissecting the Mechanisms of Immune Checkpoint Therapy. Nat Rev Immunol (2020) 20:75–6. 10.1038/s41577-020-0275-8 31925406

[51] Badoual C Hans S Merillon N Van Ryswick C Ravel P Benhamouda N. PD-1-Expressing Tumor-Infiltrating T Cells Are a Favorable Prognostic Biomarker in HPV-Associated Head and Neck Cancer. Cancer Res (2013) 73:128–38. 10.1158/0008-5472.CAN-12-2606 23135914

[52] Vermaelen K. Vaccine Strategies to Improve Anti-Cancer Cellular Immune Responses. Front Immunol (2019) 10:8. 10.3389/fimmu.2019.00008 30723469 PMC6349827

[53] Cheng YS Xu F. Anticancer Function of Polyinosinic-Polycytidylic Acid. Cancer Biol Ther (2010) 10:1219–23. 10.4161/cbt.10.12.13450 20930504

[54] Salmon H Idoyaga J Rahman A Leboeuf M Remark R Jordan S. Expansion and Activation of CD103^+^ Dendritic Cell Progenitors at the Tumor Site Enhances Tumor Responses to Therapeutic PD-L1 and BRAF Inhibition. Immunity (2016) 44:924–38. 10.1016/j.immuni.2016.03.012 PMC4980762 27096321

[55] Gilfillan CB Kuhn S Baey C Hyde EJ Yang J Ruedl C. Clec9A^+^ Dendritic Cells Are Not Essential for Antitumor CD8^+^ T Cell Responses Induced by Poly I:C Immunotherapy. J Immunol (2018) 200:2978–86. 10.4049/jimmunol.1701593 29507107

[56] Hammerich L Marron TU Upadhyay R Svensson-Arvelund J Dhainaut M Hussein S. Systemic Clinical Tumor Regressions and Potentiation of PD1 Blockade With in Situ Vaccination. Nat Med (2019) 25:814–24. 10.1038/s41591-019-0410-x 30962585

[57] Overwijk WW. Cancer Vaccines in the Era of Checkpoint Blockade: The Magic Is in the Adjuvant. Curr Opin Immunol (2017) 47:103–9. 10.1016/j.coi.2017.07.015 28806603

[58] Sikora AG Jaffarzad N Hailemichael Y Gelbard A Stonier SW Schluns KS. IFN-Alpha Enhances Peptide Vaccine-Induced CD8^+^ T Cell Numbers, Effector Function, and Antitumor Activity. J Immunol (2009) 182:7398–407. 10.4049/jimmunol.0802982 PMC2774140 19494262

[59] Pantel A Teixeira A Haddad E Wood EG Steinman RM Longhi MP. Direct Type I IFN But Not MDA5/TLR3 Activation of Dendritic Cells Is Required for Maturation and Metabolic Shift to Glycolysis After Poly IC Stimulation. PloS Biol (2014) 12:e1001759. 10.1371/journal.pbio.1001759 24409099 PMC3883643

[60] Jaks E Gavutis M Uzé G Martal J Piehler J. Differential Receptor Subunit Affinities of Type I Interferons Govern Differential Signal Activation. J Mol Biol (2007) 366:525–39. 10.1016/j.jmb.2006.11.053 17174979

[61] de Weerd NA Vivian JP Nguyen TK Mangan NE Gould JA Braniff SJ. Structural Basis of a Unique Interferon-β Signaling Axis Mediated via the Receptor IFNAR1. Nat Immunol (2013) 14:901–7. 10.1038/ni.2667 23872679

[62] Kalie E Jaitin DA Podoplelova Y Piehler J Schreiber G. The Stability of the Ternary Interferon-Receptor Complex Rather Than the Affinity to the Individual Subunits Dictates Differential Biological Activities. J Biol Chem (2008) 283:32925–36. 10.1074/jbc.M806019200 18801736

[63] François-Newton V Magno de Freitas Almeida G Payelle-Brogard B Monneron D Pichard-Garcia L Piehler J. USP18-Based Negative Feedback Control Is Induced by Type I and Type III Interferons and Specifically Inactivates Interferon α Response. PloS One (2011) 6:e22200. 10.1371/journal.pone.0022200 21779393 PMC3136508

[64] Wylie B Read J Buzzai AC Wagner T Troy N Syn G. CD8^+^XCR1^neg^ Dendritic Cells Express High Levels of Toll-Like Receptor 5 and a Unique Complement of Endocytic Receptors. Front Immunol (2018) 9:2990. 10.3389/fimmu.2018.02990 30700986 PMC6343586

[65] Hildner K Edelson BT Purtha WE Diamond M Matsushita H Kohyama M. Batf3 Deficiency Reveals a Critical Role for CD8alpha^+^ Dendritic Cells in Cytotoxic T Cell Immunity. Science (2008) 322:1097–100. 10.1126/science.1164206 PMC2756611 19008445

[66] Wiesel M Crouse J Bedenikovic G Sutherland A Joller N Oxenius A. Type-I IFN Drives the Differentiation of Short-Lived Effector CD8+ T Cells *In Vivo* . Eur J Immunol (2012) 42:320–9. 10.1002/eji.201142091 22102057

[67] Greyer M Whitney PG Stock AT Davey GM Tebartz C Bachem A. T Cell Help Amplifies Innate Signals in CD8^+^ DCs for Optimal CD8^+^ T Cell Priming. Cell Rep (2016) 14:586–97. 10.1016/j.celrep.2015.12.058 26774484

[68] Schumacher TN Schreiber RD. Neoantigens in Cancer Immunotherapy. Science (2015) 348:69–74. 10.1126/science.aaa4971 25838375

[69] Syn NL Teng MWL Mok TSK Soo RA. De-Novo and Acquired Resistance to Immune Checkpoint Targeting. Lancet Oncol (2017) 18:e731–41. 10.1016/S1470-2045(17)30607-1 29208439

[70] Fares CM Van Allen EM Drake CG Allison JP Hu-Lieskovan S. Mechanisms of Resistance to Immune Checkpoint Blockade: Why Does Checkpoint Inhibitor Immunotherapy Not Work for All Patients? Am Soc Clin Oncol Educ Book (2019) 39:147–64. 10.1200/EDBK_240837 31099674

[71] Eggermont AM Suciu S Testori A Santinami M Kruit WH Marsden J. Long-Term Results of the Randomized Phase III Trial EORTC 18991 of Adjuvant Therapy With Pegylated Interferon Alfa-2b Versus Observation in Resected Stage III Melanoma. J Clin Oncol (2012) 30:3810–8. 10.1200/JCO.2011.41.3799 23008300

