# Supplementary material for: IFNβ Is a Potent Adjuvant for Cancer Vaccination Strategies
Source: Front Immunol. 2021 Sep 6;12:735133. doi: 10.3389/fimmu.2021.735133 (PMC8450325; doi:10.3389/fimmu.2021.735133)
Supplement: Supplementary file 1 [file DataSheet_1.pdf]

Supplementary Material

1 Supplementary Figures

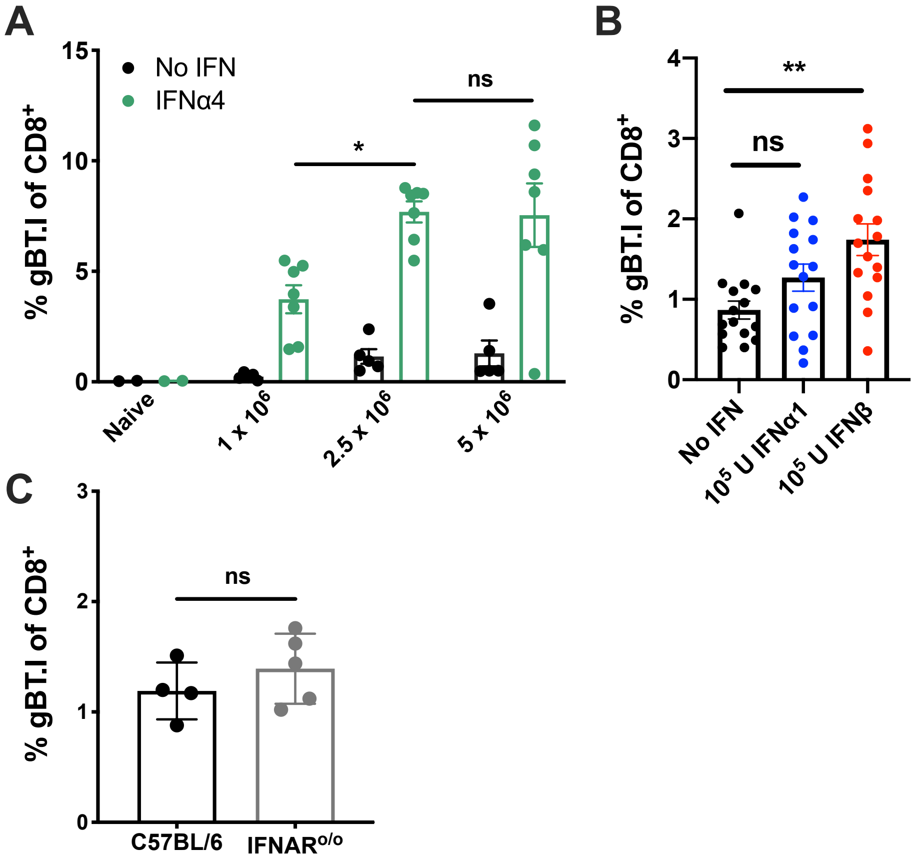

Supplementary Figure 1. Optimisation and validation of vaccination strategy. A,B C57BL/6 mice received 5 x 10^4 naïve gBT.I cells one day prior to vaccination with irradiated B16.Kb^{loss}.gB±IFN cells. Mice received titrated amounts of B16.Kb^{loss}.gB or B16.Kb^{loss}.gB_IFNα4 (n = 2-7 per group, from 1-2 independent repeats) (**A**) or 2.5 x 10^6 B16.Kb^{loss}.gB cells with or without 10^5 U recombinant IFNα1 or IFNβ (n = 15 per group from 4 independent repeats) (**B**). Seven days post-vaccination, spleens were harvested and expansion of gBT.I cells was measured. **C** 2.5 x 10^6 naïve gBT.I cells were passively transferred into C57BL/6 and IFNAR$^{o/o}$ mice (n = 4-5 per group). Splenocytes were analysed thiry days post-transfer for the presence of gBT.I cells. Data is analysed by two-way ANOVA (**A**), one-way ANOVA (**B**) or unpaired t-test (**C**), **p < 0.01, ns = not significant (p > 0.05). Bars represent mean ± SEM.

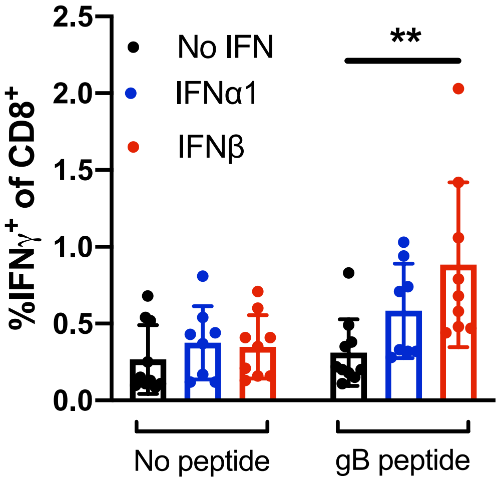

Supplementary Figure 2. Increase in tumor-specific CD8$^+$ T cells mediated by IFNβ is present at 60 days post-vaccination. C57BL/6 mice were harvested sixty days post-vaccination with 2.5 x10^6 irradiated B16.Kb^{loss}.gB±IFN cells (n = 8-9 per group) and IFNγ-producing gB-specific CD8$^+$ T cells were measured. Data is pooled from 2 independent repeats and analysed by two-way ANOVA, **$p < 0.01$. Bars represent mean ± SEM.

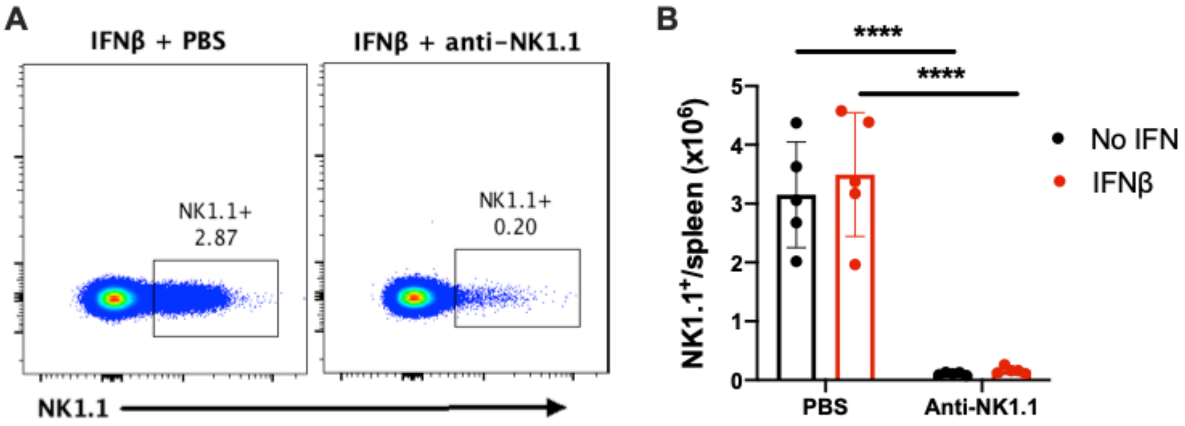

Supplementary Figure 3. Anti-NK1.1 treatment results in successful depletion of NK cells. A,B Representative flow plots of NK1.1 expression (**A**) and enumeration of $NK1.1^+$ cells (**B**) in the spleen of mice vaccinated seven days prior with 2.5 x 10^6 irradiated B16.Kb^{loss}.gB±IFNβ cells. Mice either received control PBS or 200 µg anti-NK1.1 one day prior and post vaccination (n = 5 per group). Asterisks indicate significant difference by two-way ANOVA, ****p < 0.001. Bars represent mean ± SEM.

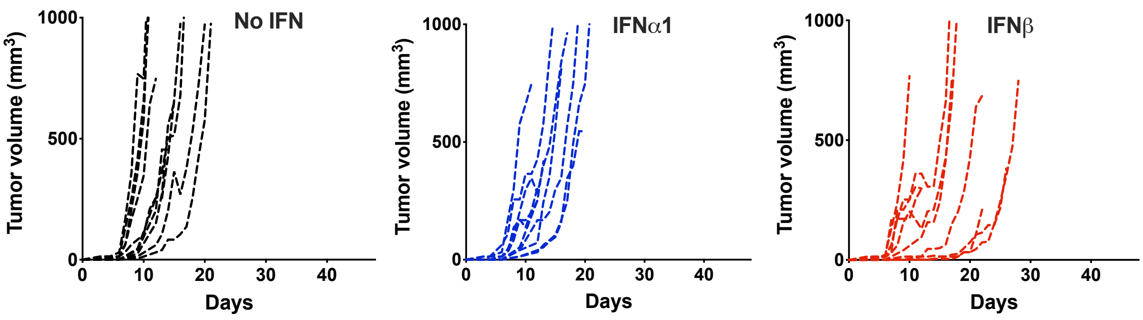

Supplementary Figure 4. Vaccination with IFNβ delays tumor progression. Individual tumor growth of individual C57BL/6 mice vaccinated with 2.5 x 10^6 irradiated B16.Kb^{loss}.gB±IFN cells three days post-B16.gB tumor inoculation (n = 10 per group from two independent experiments).

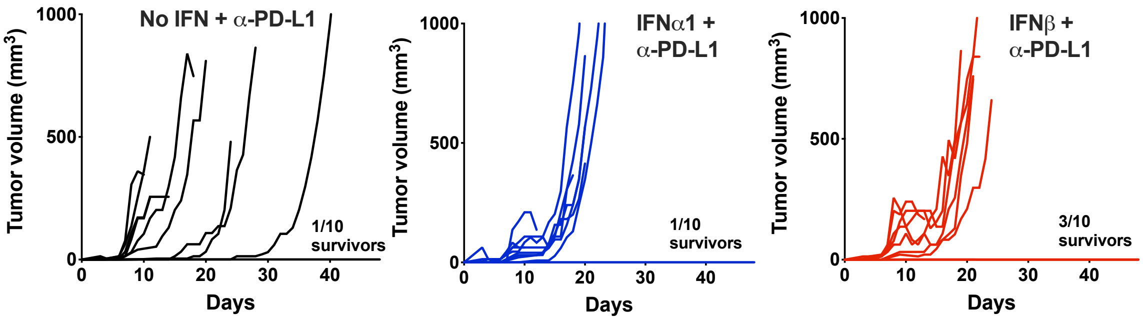

Supplementary Figure 5. Vaccination with IFNβ synergises with anti-PD-L1 checkpoint blockade therapy to increase overall survival. Individual tumor growth of C57BL/6 mice vaccinated with 2.5 x 10^6 irradiated B16.Kb^{loss}.gB±IFN cells three days post-B16.gB tumor inoculation. Mice recieved α-PD-L1 on days six, nine and twelve post-challenge (n = 10 per group from two independent experiments).

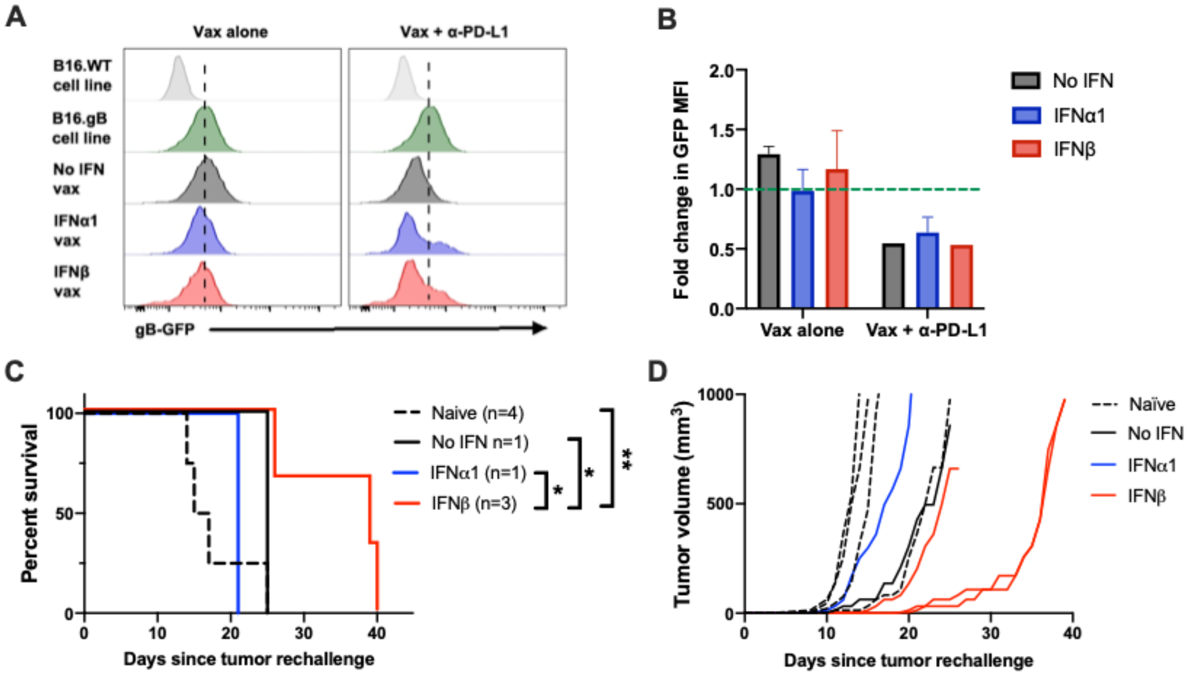

Supplementary Figure 6. Anti-PDL1 may promote tumor antigen downregulation compared to vaccination alone, and increased epitope spreading when combined with vaccination plus IFNβ. Mice were vaccinated with 2.5 x 10^6 irradiated B16.Kb^{loss}.gB±IFN cells three days post-B16.gB tumor inoculation, with some mice receiving three doses of anti-PD-L1 on days six, nine and twelve post-challenge. **(A)** Representative histograms of gB-GFP expression and **(B)** fold decrease in GFP MFI of tumors harvested at endpoint (n = 1-4 per group). **(C)** Survival and **(D)** individual growth curves of surviving mice rechallenged with B16 wildtype tumors sixty days post-initial tumor inoculation (n = 1-4 per group). Significance was assessed by Log-rank Mantel-Cox test, $**p < 0.05$, $*p < 0.1$. Bars represent mean ± SEM.
